# Supplementary material for: Membraneless channels sieve cations in ammonia-oxidizing marine archaea
Source: Nature. 2024 May 29;630(8015):230–6. doi: 10.1038/s41586-024-07462-5 (PMC11153153; doi:10.1038/s41586-024-07462-5)
Supplement: Supplementary file 1 — Supplementary Figs. 1–3 and Supplementary Tables 1–3. [file 41586_2024_7462_MOESM1_ESM.pdf]

nature portfolio

https://doi.org/10.1038/s41586-024-07462-5

Supplementary information

Membraneless channels sieve cations in ammonia-oxidizing marine archaea

In the format provided by the authors and unedited

Supplementary information for article titled

Membrane-less channels sieve cations in ammonia-oxidising marine archaea

Author information

Andriko von Kügelgen[1,2], C. Keith Cassidy[3], Sofie van Dorst[2], Lennart L. Pagani[2], Christopher Batters[4], Zephyr Ford[2], Jan Löwe[1], Vikram Alva[5], Phillip J. Stansfeld[6], and Tanmay A. M. Bharat[1,*]

Affiliations

1 – Structural Studies Division, MRC Laboratory of Molecular Biology, Francis Crick Avenue, Cambridge CB2 0QH, United Kingdom

2 – Sir William Dunn School of Pathology, University of Oxford, Oxford OX1 3RE, United Kingdom

3 – Department of Physics and Astronomy, University of Missouri-Columbia, Columbia, MO, 65211, USA

4 – Protein and Nucleic Acid Chemistry Division, MRC Laboratory of Molecular Biology, Francis Crick Avenue, Cambridge CB2 0QH, United Kingdom

5 – Department of Protein Evolution, Max Planck Institute for Biology Tübingen, Max-Planck-Ring 5, Tübingen 72076, Germany

6 – School of Life Sciences and Department of Chemistry, Gibbet Hill Campus, University of Warwick, Coventry CV4 7AL, United Kingdom

*** Correspondence to**

Tanmay A. M. Bharat, email: tbharat@mrc-lmb.cam.ac.uk

Supplementary Figures

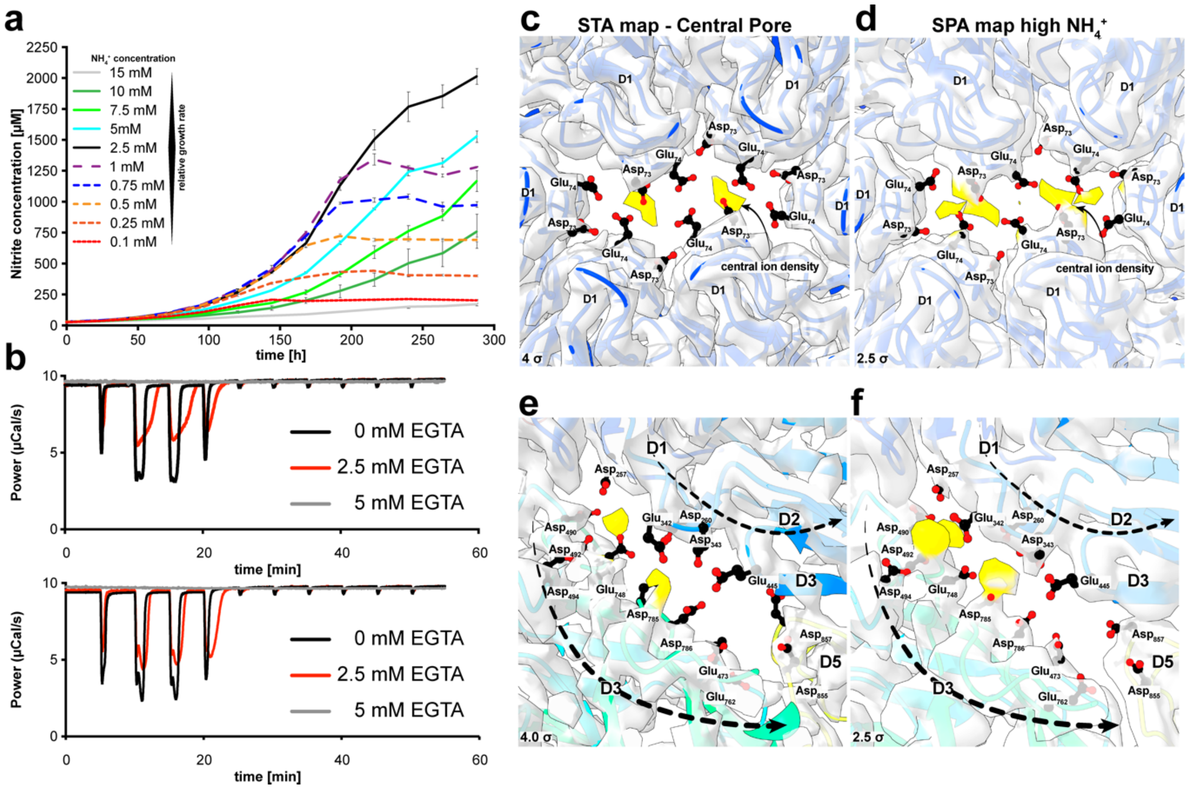

Supplementary Fig. 1| Ammonium growth, ITC and cryo-EM at increased ammonium concentration.

a, Growth curves of *N. maritimus* in differing ammonium concentrations. Different curves are shown in different colour (colour bar is shown on the left). Growth curves run as biological triplicates with one out of three replicates shown. Error bars are displayed as standard error of mean (S.E.M.) **b**, Ammonium binding to *N. maritimus* cells, biological repeats are shown at different EGTA concentrations, which show different levels of S-layer disruption. **c**-**f**, Close-up views of pore regions of STA (**c**,**e**) and SPA maps with high ammonium concentration (**d**,**f**) with additional unexplained densities.

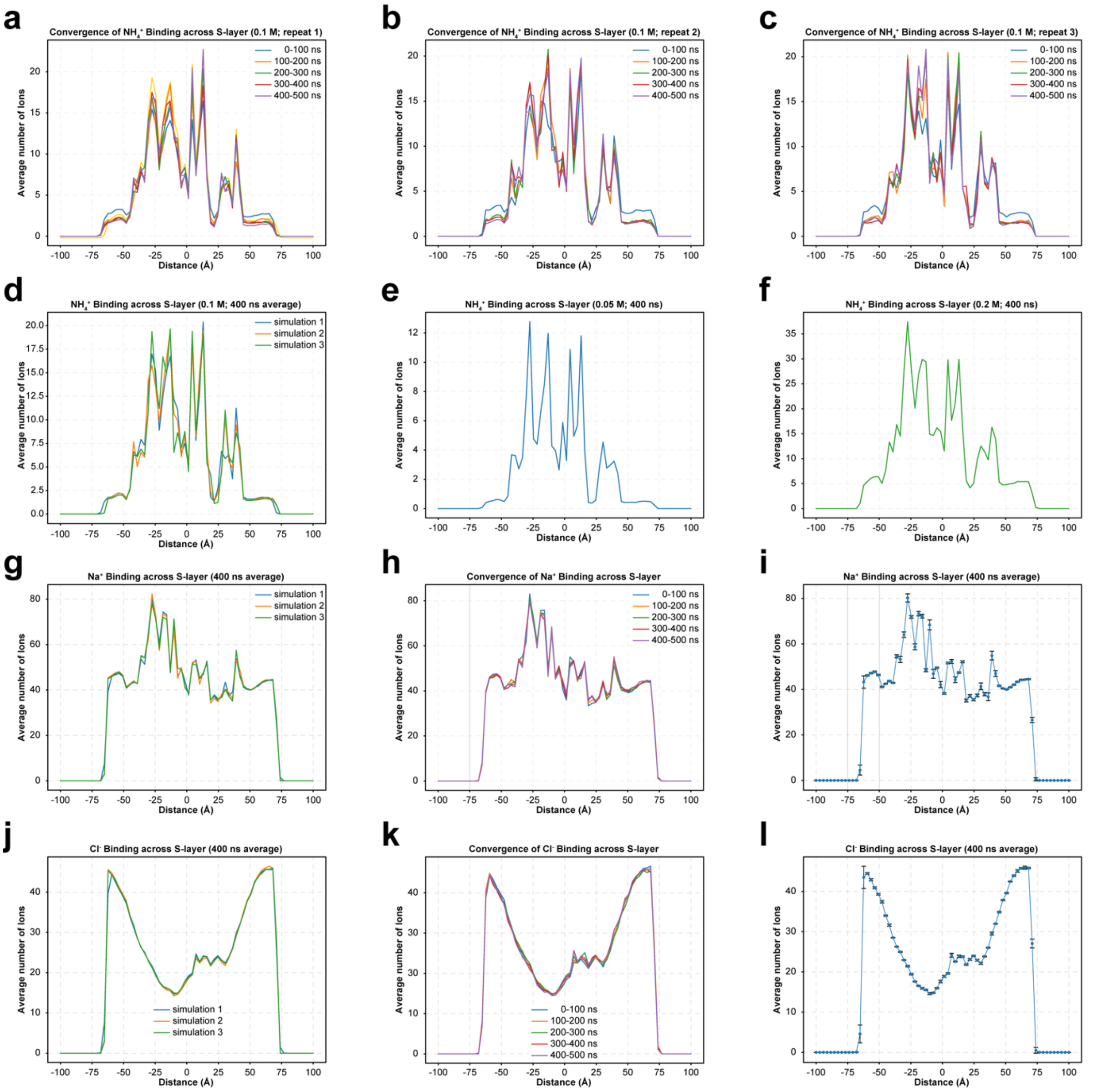

Supplementary Fig. 2| Ionic distributions through the S-layer in MD simulations.

a-c, Averaged residence of ammonium (NH_4^+) ions through (in the directional orthogonal to) the S-layer in MD simulations, from the membrane proximal side (left) to the extracellular side (right) across different time windows showing convergence of the MD simulations. **d,** Overlay of averaged residence of ammonium (NH_4^+) ions in three independent simulations averaged over the last 400 ns of each simulation. Note that the first 100 ns of each simulation is not included to allow for equilibration (see Methods). **e-f,** Averaged residence of ammonium (NH_4^+) ions through the S-layer in MD simulations at **e,** reduced ammonium concentrations (0.05 M) and **f,** increased ammonium concentrations (0.2 M) **g,** Overlay of averaged residence

of sodium (Na^+) ions in three independent simulations averaged over the last 400 ns of each simulation. **h,** Convergence of sodium ion residence in MD-simulation (one out of three replicates is shown). **i,** Averaged residence of sodium (Na^+) ions in three independent simulations shown in (g) averaged over the last 400 ns of each simulation. Errors bars denote ±1 standard deviation. **j,** Overlay of averaged residence of chloride (Cl^-) ions across three independent simulations averaged over the last 400 ns of each simulation. **k,** Convergence of chloride ion residence in MD-simulations (one out of three replicates is shown). **l,** Averaged residence of chloride (Cl^-) ions in three independent simulations shown in (j) averaged over the last 400 ns of each simulation. Errors bars denote ±1 standard deviation.

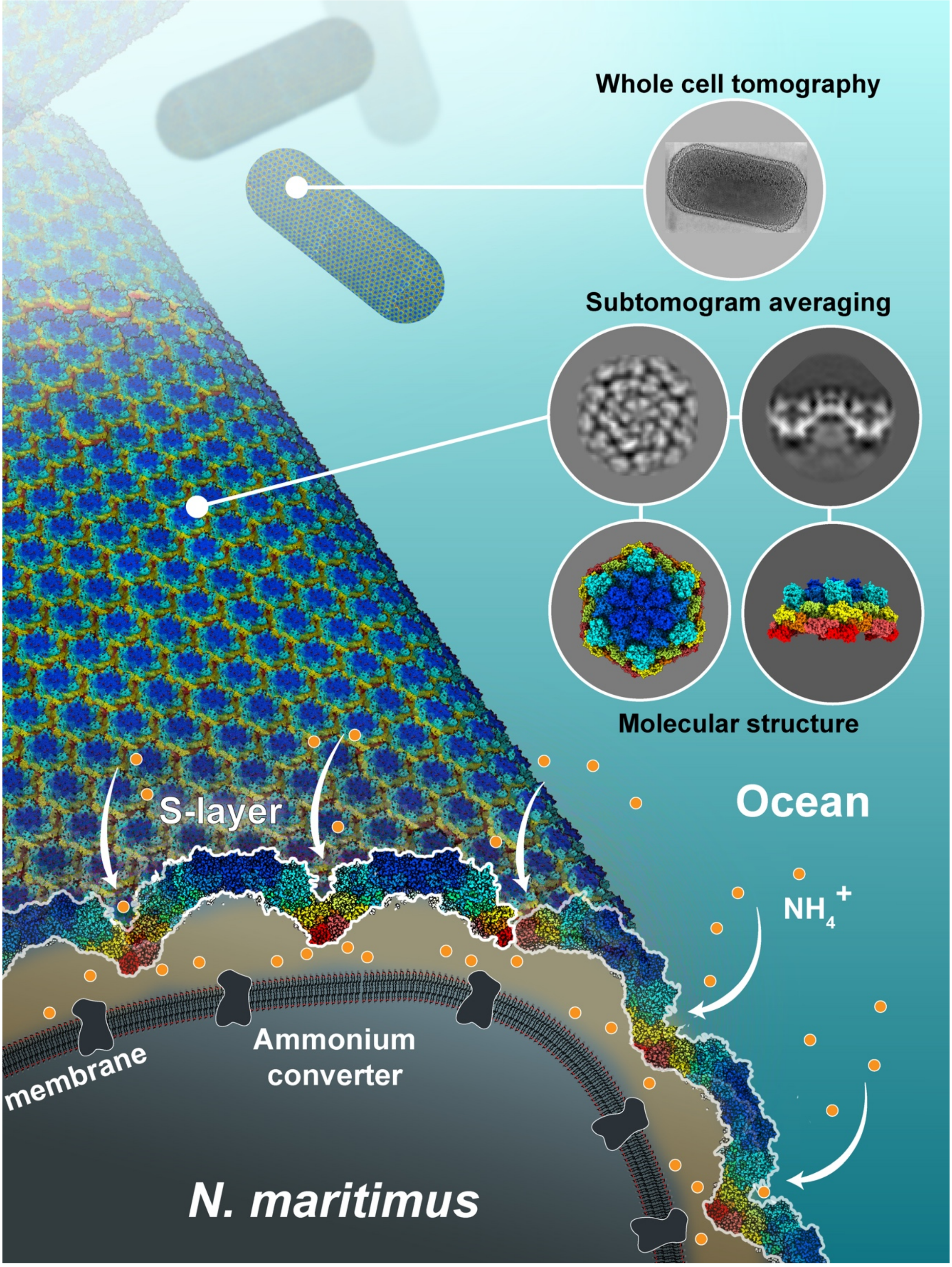

Supplementary Fig. 3| The S-layer acts as a multi-channel ammonium sieve in marine archaea.

Schematic description of the methodology used and results generated in this study. Direct structure determination from intact cells allowed us to study a biogeochemically important

process *in situ*, related to ammonium entrapment and multi-path ammonium channelling aided by gradual decrease in charge along the *N. maritimus* S-layer.

Supplementary Tables

Supplementary Table 1| Predicted net charges of S-layer proteins

Organism	Protein	NCBI/Uniprot ID	Charge at pH=7.4
**Nitrososphaerota (genus Nitrosopumilus)**			
Nitrosopumilus maritimus	SlaA	A9A4Y9	-211.7
Nitrosopumilus maritimus	SlaA1	A9A5U2	-213.7
Nitrosopumilus maritimus	SlaB	A9A4Y8	-20
Ca. Nitrosomarinus catalina	SlaA	WP_086907064.1	-206.6
Ca. Nitrosomarinus catalina	SlaB	WP_225971295.1	-19.1
Ca. Nitrosopumilus sediminis	SlaA	WP_014965859.1	-194.5
Ca. Nitrosopumilus sediminis	SlaA1	WP_016940265.1	-67.2
Ca. Nitrosopumilus sediminis	SlaB	AFS83491.1	-17.1
Ca. Nitrosopumilus salaria	SlaA	WP_048097520.1	-184.5
Ca. Nitrosopumilus salaria	SlaB	WP_008300022.1	-18.1
Nitrosopumilus adriaticus	SlaA	WP_048115011.1	-197.5
Nitrosopumilus adriaticus	SlaA1	WP_048116094.1	-70.2
Nitrosopumilus adriaticus	SlaB	WP_048115014.1	-14.1
Nitrosopumilus piranensis	SlaA	WP_148702275.1	-232.8
Nitrosopumilus piranensis	SlaA1	AJM93167.1	-63.3
Nitrosopumilus piranensis	SlaB	WP_148702274.1	-16.1
Ca. Nitrosopumilus koreensis	SlaA	WP_014963920.1	-202.8
Ca. Nitrosopumilus koreensis	SlaA1	AFS80276.1	-69.2
Ca. Nitrosopumilus koreensis	SlaB	WP_014963919.1	-16
**Nitrososphaerota (genus Cenarchaeum)**			
Cenarchaeum symbiosum A	SlaA	ABK76806.1	-285.2
Cenarchaeum symbiosum A	SlaA1	ABK76804.1	-284.3
Cenarchaeum symbiosum A	SlaA2	ABK78662.1	-277.4
Cenarchaeum symbiosum A	SlaB	ABK78037.1	-25.9
**Nitrososphaerota (genus Nitrosarchaeum)**			
Ca. Nitrosarchaeum limnium	SlaA	EGG41144.1	-130.1
Ca. Nitrosarchaeum limnium	SlaB	WP_010193228.1	-13.2
Nitrosarchaeum koreense	SlaA	WP_048110047.1	-173.6
Nitrosarchaeum koreense	SlaB	WP_007551396.1	-22.1
**Nitrososphaerota (genus *Ca.* Nitrosotenuis)**			
Ca. Nitrosotenuis aquarius	SlaA	WP_100183187.1	-144.1
Ca. Nitrosotenuis aquarius	SlaB	WP_100183188.1	-17.1
Ca. Nitrosotenuis uzonensis	SlaA	WP_048196670.1	-138.1
Ca. Nitrosotenuis uzonensis	SlaB	WP_048196668.1	-12.1
Ca. Nitrosotenuis cloacae	SlaA	WP_048188611.1	-163.1
Ca. Nitrosotenuis cloacae	SlaB	AJZ75755.1	-16.2
Ca. Nitrosotenuis chungbukensis	SlaA	WP_042684620.1	-150.9
Ca. Nitrosotenuis chungbukensis	SlaB	WP_042684623.1	-11.2
**Nitrososphaerota (genus *Ca.* Nitrosotalea)**			

Ca. Nitrosotalea devanaterra	SlaA	CUR50924.1	-64.3
Ca. Nitrosotalea devanaterra	SlaB	CUR51008.1	-3.2
Ca. Nitrosotalea okcheonensis	SlaA	WP_157928030.1	-58.3
Ca. Nitrosotalea okcheonensis	SlaB	WP_231911788.1	-1.1
Ca. Nitrosotalea sinensis	SlaA	WP_101008924.1	-63.3
Ca. Nitrosotalea sinensis	SlaB	WP_245871850.1	-3.2
Ca. Nitrosotalea bavarica	SlaA	WP_101477936.1	-77
Ca. Nitrosotalea bavarica	SlaB	WP_101477185.1	-9.2
**Nitrososphaerota (genus Nitrososphaera)**			
Nitrososphaera viennensis	SlaA	A0A060HS03	-45.8
Nitrososphaera viennensis	SlaB	WP_144239588.1	-3.2
Ca. Nitrososphaera evergladensis	SlaA	WP_148699684.1	-39.1
Ca. Nitrososphaera evergladensis	SlaB	WP_148699685.1	-3.2
Ca. Nitrososphaera gargensis	SlaA	NC_018719.1_80	-69.7
Ca. Nitrososphaera gargensis	SlaB	WP_148680754.1	-7
**Nitrososphaerota (genus *Ca.* Nitrosocaldus)**			
Ca. Nitrosocaldus cavascurensis	SlaA	WP_148695099.1	-56.6
Ca. Nitrosocaldus cavascurensis	SlaB	WP_103287785.1	-8.2
**Thermoproteota (order Sulfolobales)**			
Acidianus ambivalens	SlaA	B1GT61	-25.1
Acidianus ambivalens	SlaB	B1GT62	-25.4
Sulfolobus solfataricus	SlaA	F0NHT7	-3.4
Sulfolobus solfataricus	SlaB	Q980C6	-1.1
Sulfolobus acidocaldarius	SlaA	Q4J6E5	-11.5
Sulfolobus acidocaldarius	SlaB	AHC52676.1	3.8
Metallosphaera sedula	SlaA	A4YHQ8	-16.5
Metallosphaera sedula	SlaB	A4YHQ9	-3.2
**Thermoproteota (order Desulfurococcales)**			
Hyperthermus butylicus	SlaA	WP_011822157.1	-95.8
Hyperthermus butylicus	SlaB	ABM80840.1	-23.3
Ignisphaera aggregans	SlaA	ADM26894.1	-40.5
Ignisphaera aggregans	SlaB	ADM26895.1	-1.7
Staphylothermus marinus	Tetrabrachion	Q54436	-66.6
Aeropyrum pernix	SlaA	Q9YEG7	-180.2
**Euryarchaeota (order Haloferacales)**			
Haloferax volcanii	Csg	P25062	-138
Haloferax mediterranei	Csg	I3R2Z6	-149.2
**Euryarchaeota (order Methanomicrobiales)**			
Methanospirillum hungatei	Csg	WP_011449226.1	-34.5
**Euryarchaeota (order Methanobacteriales)**			
Methanothermus fervidus	Csg	P27373	-1.2

Supplementary Table 2 | Ammonium binding residues predicted by MD. Listed are residues with average occupancy >50% as computed by PyLipID over three 0.1 M NH_4^+ NmSLP hexamer simulations.

#	Residue	Avg Occ	std dev	#	Residue	Avg Occ	std dev
1	ASP71	50.55	10.33	32	ASP918	75.53	3.91
2	ASP73	81.03	14.89	33	GLU923	80.67	6.20
3	GLU74	90.33	15.01	34	GLU1002	95.25	2.50
4	GLU143	55.97	23.37	35	GLU1082	71.14	17.70
5	GLU196	75.17	8.16	36	ASP1088	80.25	19.95
6	ASP257	69.81	3.01	37	GLU1150	89.75	3.77
7	ASP260	76.83	8.44	38	ASP1152	81.59	3.64
8	ASP293	85.19	9.06	39	ASP1248	51.44	18.72
9	GLU294	61.70	7.26	40	ASP1263	93.56	0.88
10	GLU321	87.30	8.96	41	ASP1278	53.89	1.77
11	ASP343	83.19	8.55	42	ASP1297	94.64	3.43
12	ASN444	52.16	6.03	43	PRO1298	63.41	9.83
13	GLU445	58.80	4.19	44	GLU1299	96.55	2.14
14	GLU473	71.03	5.53	45	ASP1308	55.72	1.09
15	ASP490	79.58	5.88	46	GLU1318	73.42	4.43
16	ASP492	61.67	2.19	47	GLU1329	73.72	12.50
17	ASP494	84.39	4.57	48	GLU1352	53.44	21.28
18	ASP510	69.78	11.86	49	ASP1353	51.31	1.00
19	GLU516	67.83	11.66	50	ASP1355	74.81	7.91
20	GLU610	86.19	5.34	51	ASP1394	57.61	19.28
21	GLU746	69.72	6.08	52	GLU1486	66.89	6.87
22	GLU748	66.78	3.99	53	ASP1487	71.53	6.62
23	GLU762	87.22	3.26	54	ASP1538	52.47	0.55
24	ASP785	71.53	1.57	55	ASP1549	56.58	6.51
25	ASP786	78.22	1.19	56	ASP1572	51.19	18.72
26	GLU798	95.66	2.47	57	GLU1590	75.47	1.75
27	ASP799	83.31	0.17	58	GLU1592	76.53	5.21
28	ASP855	86.61	1.13	59	ASP1598	66.31	6.33
29	ASP857	68.42	2.07	60	ASP1604	57.92	22.45
30	ASP899	92.89	1.93	61	ASP1607	76.86	2.95
31	ASP916	75.86	3.85				

Supplementary Table 3 | Reliability and reproducibility checklist for molecular dynamics simulations.

1. Convergence of simulations and analysis

1a. Is an evaluation presented in the text to show that the property being measured has equilibrated in the simulations (e.g., time-course analysis)? **YES**

1b. Then, is it described in the text how simulations are split into equilibration and production runs and how much data were analyzed from production runs? **YES**

1c. Are there at least 3 simulations per simulation condition with statistical analysis? **YES**

1d. Is evidence provided in the text that the simulation results presented are independent of initial configuration? **YES**

2. Connection to experiments

2a. Are calculations provided that can connect to experiments (e.g., loss or gain in function from mutagenesis, binding assays, NMR chemical shifts, J-couplings, SAXS curves, interaction distances or FRET distances, structure factors, diffusion coefficients, bulk modulus and other mechanical properties, etc.)? **YES, simulations use structures derived from cryoEM experiments and simulation results are compared directly to cryoEM data.**

3. Method choice

3a. Do simulations contain membranes, membrane proteins, intrinsically disordered proteins, glycans, nucleic acids, polymers, or cryptic ligand binding? **NO**

3b. Is it described in the text whether the accuracy of the chosen model(s) is sufficient to address the question(s) under investigation (e.g., all-atom vs. coarse-grained models, fixed charge vs. polarizable force fields, implicit vs. explicit solvent or membrane, specific force field and water model, etc.? **Our chosen protocol (all-atom, explicit solvent, CHARMM36 force field) is more than adequate to characterize the interactions of interest in this study. An explicit discussion of this in the text is not required.**

3c. Is the timescale of the event(s) under investigation beyond the brute-force MD simulation timescale in this study that enhanced sampling methods are needed? **NO**

If YES, are the parameters and convergence criteria for the enhanced sampling method clearly stated? If NO, is the evidence provided in the text? **N/A**

4. Code and reproducibility

4a. Is a table provided describing the system setup that includes simulation box dimensions, total number of atoms, number of water molecules, salt concentration, lipid composition (number of molecules and type)? **All this information is provided in the Methods.**

4b. Are other parameters for the system setup described in the text, such as protonation state, type of structural restraints if applied, nonbonded cutoff, thermostat and barostat, etc.? **All this information is provided in the Methods.**

4c. Is it described in the text what simulation and analysis software and which versions are used? **All this information is provided in the Methods.**

4d. Are initial coordinate and simulation input files and a coordinate file of the final output provided as supplementary files or in a public repository? **Initial coordinates have been deposited in the Protein Data Bank. All simulation input settings have been described in the Methods.**

4e. Is there custom code or custom force field parameters? **Ammonium parameters were obtained by trivial analogy with existing parameters for methylammonium.**

If YES, are they provided as supplementary files or in a public repository? **The file is appended below as .txt**

```
* Topology File.
*
   99   1
MASS  -1  NG3P3     14.00700 ! primary NH3+, phosphatidylethanolamine
MASS  -1  HGP2       1.00800 ! polar H, +ve charge

RESI AMMO  1.000
GROUP
ATOM NZ  NG3P3    -0.32 !         HZ1
ATOM HZ1 HGP2      0.33 !      (+) |
ATOM HZ2 HGP2      0.33 !    HZ4---NZ--HZ2
ATOM HZ3 HGP2      0.33 !          |
ATOM HZ4 HGP2      0.33 !         HZ3

BOND NZ     HZ1                   ! dist        1.0400
BOND NZ     HZ2          ! dist        1.0400
BOND NZ     HZ3                   ! dist        1.0400
BOND NZ     HZ4                   ! dist        1.0400

##########################################################################

* Force Field Parameter File.
*

ATOMS
MASS  -1  NG3P3     14.00700 ! primary NH3+, phosphatidylethanolamine
MASS  -1  HGP2       1.00800 ! polar H, +ve charge

BONDS
NG3P3  HGP2    403.00     1.0400 ! PROT new stretch and bend; methylammonium (KK 03/10/92)

ANGLES
HGP2   NG3P3  HGP2     44.00    109.50 ! PROT new stretch and bend; methylammonium (KK
03/10/92)

DIHEDRALS

IMPROPER


NONBONDED nbxmod  5 atom cdiel shift vatom vdistance vswitch -
cutnb 14.0 ctofnb 12.0 ctonnb 10.0 eps 1.0 e14fac 1.0 wmin 1.5
!              Emin     Rmin/2             Emin/2     Rmin  (for 1-4's)
!           (kcal/mol)    (A)
```

Supplementary Video Legends

Supplementary Video 1| Cryo-electron tomography of *N. maritimus* and NmSLP S-layer structure.

The cryo-ET STA map and structure of the *N. maritimus* S-layer show how NmSLP monomers form the lattice. Different views of the S-layer are shown with text annotations.

Supplementary Video 2| Cryo-electron tomography of *N. maritimus*.

Slices through tomogram of the same *N. maritimus* cell shown in Supplementary Video 1 without segmentation to enable direct assessment of the cryoET density.

Supplementary Video 3| Cryo-EM structure of isolated NmSLP and structural comparison to cryo-ET structure.

The cryo-EM map and structure of the *N. maritimus* S-layer compared with the *in situ* STA structure.

Supplementary Video 4| Ammonium binding in MD simulations.

Residues identified by PyLipID with average ammonium occupancy >50% for three 0.1 M NH_4^+ NmSLP hexamer simulations. Each residue is coloured by its occupancy value, which was averaged over the six NmSLP monomers, and mapped on to the hexamer structure. Residue numbers and occupancies with standard deviations are given in Supplementary Table 2. This video was produced using the VDM software.
