# Supplementary material for: Membraneless channels sieve cations in ammonia-oxidizing marine archaea
Source: Nature. 2024 May 29;630(8015):230–6. doi: 10.1038/s41586-024-07462-5 (PMC11153153; doi:10.1038/s41586-024-07462-5)
Supplement: Supplementary file 2 — Reporting Summary [file 41586_2024_7462_MOESM2_ESM.pdf]

nature portfolio

Corresponding author(s): Tanmay A.M. Bharat

Last updated by author(s): Apr 4, 2024

Reporting Summary

Nature Portfolio wishes to improve the reproducibility of the work that we publish. This form provides structure for consistency and transparency in reporting. For further information on Nature Portfolio policies, see our Editorial Policies and the Editorial Policy Checklist.

Statistics

For all statistical analyses, confirm that the following items are present in the figure legend, table legend, main text, or Methods section.

n/a	Confirmed	
☐	☒	The exact sample size (*n*) for each experimental group/condition, given as a discrete number and unit of measurement
☐	☒	A statement on whether measurements were taken from distinct samples or whether the same sample was measured repeatedly
☐	☒	The statistical test(s) used AND whether they are one- or two-sided<br>*Only common tests should be described solely by name; describe more complex techniques in the Methods section.*
☒	☐	A description of all covariates tested
☒	☐	A description of any assumptions or corrections, such as tests of normality and adjustment for multiple comparisons
☐	☒	A full description of the statistical parameters including central tendency (e.g. means) or other basic estimates (e.g. regression coefficient) AND variation (e.g. standard deviation) or associated estimates of uncertainty (e.g. confidence intervals)
☐	☒	For null hypothesis testing, the test statistic (e.g. *F*, *t*, *r*) with confidence intervals, effect sizes, degrees of freedom and *P* value noted<br>*Give P values as exact values whenever suitable.*
☒	☐	For Bayesian analysis, information on the choice of priors and Markov chain Monte Carlo settings
☒	☐	For hierarchical and complex designs, identification of the appropriate level for tests and full reporting of outcomes
☒	☐	Estimates of effect sizes (e.g. Cohen's *d*, Pearson's *r*), indicating how they were calculated

Our web collection on statistics for biologists contains articles on many of the points above.

Software and code

Policy information about availability of computer code

Data collection	EPU v.2+, SerialEM v.3 and v.4+
Data analysis	RELION v3.1, RELION v4.0, CTFFIND v4.1, TOPAZ v.0.2.5, IMOD v4.9.13, COOT v0.9.6, ChimeraX v1.5, Chimera v1.16, PyMOL v2.5.2, CCPEM v1.6.0, Refmac5 (Servalcat v0.2.85), PHENIX v.1.19-4092-000, VDM v.1.94,, NAMD v2.14, MATLAB v.R2019b, BLAST v.2.13.0+, HHpred v.57c87071, AlphaFold v.2.2.0, SignalP v.6.0, EMBOSS 6.6.0.0

For manuscripts utilizing custom algorithms or software that are central to the research but not yet described in published literature, software must be made available to editors and reviewers. We strongly encourage code deposition in a community repository (e.g. GitHub). See the Nature Portfolio guidelines for submitting code & software for further information.

Data

Policy information about availability of data

All manuscripts must include a data availability statement. This statement should provide the following information, where applicable:

- Accession codes, unique identifiers, or web links for publicly available datasets
- A description of any restrictions on data availability
- For clinical datasets or third party data, please ensure that the statement adheres to our policy

Maps have be deposited in the Electron Microscopy Data Bank (EMDB) and atomic coordinates have been deposited in the Protein Data Bank (PDB). The accession codes are: In vitro S-layer structure (single particle analysis, SPA) with two-fold symmetry (C2): PDB ID 8C8L, EMD-16483; In vitro S-layer structure (SPA) with two-

fold symmetry (C6): PDB ID 8C8K, EMD-16482; In vitro S-layer structure (SPA) composite map: PDB ID 8C8M, EMD-16484; In vitro S-layer structure (SPA) with high NH_4Cl and two-fold symmetry (C2): EMD-16486; In situ S-layer structure (subtomogram averaging, STA) with two-fold symmetry (C2): PDB ID 8C8N, EMD-16487; In situ S-layer structure (STA) with two-fold symmetry (C6): PDB ID 8C8O, EMD-16489; In situ S-layer structure (STA) composite map: PDB ID 8C8R, EMD-16492; For more details see Extended Data tables 1 and 2, respectively. No new sequences are reported in this study.

Research involving human participants, their data, or biological material

Policy information about studies with human participants or human data. See also policy information about sex, gender (identity/presentation), and sexual orientation and race, ethnicity and racism.

Reporting on sex and gender	Not applicable
Reporting on race, ethnicity, or other socially relevant groupings	Not applicable
Population characteristics	Not applicable
Recruitment	Not applicable
Ethics oversight	Not applicable

Note that full information on the approval of the study protocol must also be provided in the manuscript.

Field-specific reporting

Please select the one below that is the best fit for your research. If you are not sure, read the appropriate sections before making your selection.

☒ Life sciences ☐ Behavioural & social sciences ☐ Ecological, evolutionary & environmental sciences

For a reference copy of the document with all sections, see nature.com/documents/nr-reporting-summary-flat.pdf

Life sciences study design

All studies must disclose on these points even when the disclosure is negative.

Sample size	Cryo-EM and cryo-ET data set sizes were selected to obtain high-resolution reconstructions. The sample size was chosen to reach a resolution of 3.3-4.5 Å for the cryoET data and 2.7 Å for cryoEM data.
Data exclusions	Cryo-EM micrographs and tilt-series were selected based on high resolution content in the cryo-EM or cryo-ET workflow. Extracted particles not suitable for high-resolution reconstruction were excluded during the processing. For further details on image selection see Extended Data Tables 1 and 2.
Replication	The structures were solved as per the accepted protocols for data analysis, including an unbiased Fourier Shell correlation of independently aligned and averaged halves of the data. Triplicate experiments were performed for molecular simulations and growth curves, all replicates showed the same results. Other experiments like ITC were performed in triplicates. All replicates showed similar results.
Randomization	Not relevant to this study. Randomisation was not needed for the statistics.
Blinding	Not relevant to this study. Not needed for the statistics.

Reporting for specific materials, systems and methods

We require information from authors about some types of materials, experimental systems and methods used in many studies. Here, indicate whether each material, system or method listed is relevant to your study. If you are not sure if a list item applies to your research, read the appropriate section before selecting a response.

Materials & experimental systems

n/a	Involved in the study
☒	☐ Antibodies
☒	☐ Eukaryotic cell lines
☒	☐ Palaeontology and archaeology
☒	☐ Animals and other organisms
☒	☐ Clinical data
☒	☐ Dual use research of concern
☒	☐ Plants

Methods

n/a	Involved in the study
☒	☐ ChIP-seq
☒	☐ Flow cytometry
☒	☐ MRI-based neuroimaging

Plants

Seed stocks	Not relevant to this study.
Novel plant genotypes	Not relevant to this study.
Authentication	Not relevant to this study.
